# Recommendations for reporting regression-based norms and the development of free-access tools to implement them in practice

**DOI:** 10.1371/journal.pone.0325770

**Published:** 2025-06-23

**Authors:** Rok Blagus, Bojan Leskošek, Francisco B. Ortega, Grant Tomkinson, Gregor Jurak

**Affiliations:** 1 Institute for Biostatistics and Medical Informatics, University of Ljubljana, Ljubljana, Slovenia; 2 Faculty of Mathematics, Natural Sciences and Information Technologies, University of Primorska, Koper, Slovenia; 3 Faculty of Sport, University of Ljubljana, Ljubljana, Slovenia; 4 Department of Physical Education and Sports, Faculty of Sport Sciences, Sport and Health University Research Institute (iMUDS), University of Granada, and CIBEROBN Physiopathology of Obesity and Nutrition, Granada, Spain; 5 Faculty of Sport and Health Sciences, University of Jyväskylä, Jyväskylä, Finland; 6 Alliance for Research in Exercise, Nutrition and Activity (ARENA), Allied Health and Human Performance, University of South Australia, Adelaide, South Australia, Australia; Universiti Utara Malaysia, MALAYSIA

## Abstract

Norm-referenced tests compare individuals to a reference or source population. Norms usually depend on individual characteristics (norm-predictors) like age, gender, etc. Regression-based norming, a type of continuous norming, allows for exact evaluation of the test-taker’s score for any combination of the norm-predictors. Regression-based norms are often presented in tables and graphs in scientific papers, where only selected centiles for some combination of norm-predictors are summarized. Therefore exact score evaluation for any combination of norm-predictors is usually impossible because it requires a detailed presentation of all estimated model parameters which are usually undisclosed. Furthermore, the fitted models, like those from the R *gamlss* package, may include individual data that are usually protected by law and consent, which prevent data sharing. Thus, this paper provides recommendations for publishing regression-based norms that allow precise score evaluation for any combination of the norm-predictors while protecting participant privacy. We outline specific requirements for such publications: a) the exact presentation of the underlying fitted regression model that contains the estimates of all model parameters and other information required for exact evaluation; b) computer sharable fit of the model that does not contain any sensitive information and can be used by those with programming skills to evaluate scores; and c) a web-based application that can be used by those without programming skills to use the results of the fitted model. To facilitate publication and utilization of such regression-based norms, we have developed and provided an open-source R package of tools for authors and users alike. Following our recommendations, any user can access the underlying models while data privacy is maintained. This approach ensures broad accessibility and practical application of norms, allowing other researchers to accurately interpret their individual data against such norms.

## Introduction

In scientific work, measurement plays a crucial role in evaluating the characteristics of objects or events. Various tests (measures, instruments, or scales) are employed to gather data, and these tests can be classified as either criterion-referenced or norm-referenced [1]. Criterion-referenced tests assess performance against a pre-specified standard. In contrast, norm-referenced tests assess and compare an individual’s performance against a pre-established reference population (e.g., national, regional, or global population comparator). These norm-referenced tests are used across various fields including psychology, education, and healthcare to evaluate corresponding abilities or attributes [2]. For example, in psychology, norm-referenced tests like the Wechsler Intelligence Scale for Children (WISC) are used to assess intellectual abilities [3]. In education, standardized tests like the Scholastic Aptitude Test and American College Test help determine student placement and readiness for college [4]. Similarly, in healthcare, growth charts based on norms for height and mass are essential tools for pediatricians to monitor child development [5]. In sports, norm-referenced tests are often used for performance evaluation (e.g., fitness or skill-based profiling and monitoring) [6,7], talent identification [8], and to inform coaching strategies [9].

Norms (normative data or normative values) usually depend on individual characteristics (norm-predictors) like age, sex, and educational level in the WISC, which imply that there are multiple reference populations [10]. Continuous norming [11], where one uses the information provided by the continuous nature of the norm-predictors in the norm construction, can be used to efficiently address this. Regression-based norming [12,13], a type of continuous norming, enables one to obtain normed scores for each combination of raw score and norm-predictors values. The *gamlss* library in R, that implements Generalized Additive Models for Location, Scale, and Shape (GAMLSS) is one of the most commonly used tools for regression-based norming. For example, this highly versatile model family, suitable for a wide range of empirical norming cases, has been used to create reference centile values and curves for measures of body size in pediatric studies, allowing for more accurate assessments of child growth patterns [14], for fitness tests to monitor the motor development of children [7] and changes across the adult lifespan [15]. An important characteristic in continuous norming is that the association between the score and norm-predictors is usually non-linear, requiring appropriate modelling techniques to allow for such non-linear associations. In regression-based norming, P-splines [16], a popular version of penalized regression splines [17], are often used for this purpose. P-splines together with GAMLSS provide one of the most powerful tools in modern regression analysis [18] and if used appropriately should accommodate most practical applications. In the scientific literature, results of regression-based norming are typically tabulated and visualized while the underlying regression model is neither fully disclosed nor made available to others. Tables often display the specific centile values (e.g., 1^st^, 10^th^, 25^th^, 50^th^) for various values of the norm-predictors (e.g., age intervals 20–29y, 30–39y), while the centile curves or bands for select centiles are graphically displayed [19]. Since these resources only include select centiles for selected values (or ranges) of norm-predictors, they cannot be used to determine exact centile values for any combination of the raw score and norm-predictor values [20], thus violating the original purpose of using continuous norming. This lack of detailed data needed to calculate the exact centiles for exact values of the norm-predictors is exacerbated when additive terms, such as P-splines, are required [21]. These terms necessitate detailed model parameters and other details, which are often not fully disclosed in published papers, limiting the ability of professionals and test-takers to apply the norms accurately [20].

Thus, to enable exact normative evaluation for any combination of the raw score and norm-predictor values, the authors of the norms are recommended to fully disclose the entire underlying estimated regression model that includes all the details about the additive terms, when they are present in the model. When using the *gamlss* library to fit the underlying regression model, there is, however, a serious drawback: the authors may not be allowed to publish the obtained GAMLSS models as they contain all the individual values of variables used for generating the model, which are usually protected by law (e.g., the general data protection rule (GDPR) in the European Union) or study participants’ informed consent agreement. Publishing personal data can raise ethical issues, as it may reveal data that cause harm to study participants (e.g., reputational damage, psychological harm, or financial loss). Even though the data are published in anonymized form, there is a risk of deidentification [22]. So, even though the *gamlss* library contains functions (e.g., *centile.pred*) to easily and precisely evaluate the test-taker’s score, field professionals cannot use those functions because they do not possess the underlying regression model as the authors may not be allowed to share it.

Accordingly, this paper provides recommendations for the publication of norms that will enable the exact regression-based normative evaluation of test-taker’s score while respecting the privacy of personal data used in norms construction. We also develop and describe the tools that will enable authors to efficiently publish the norms in accordance with the recommendations and enable end-users (e.g., practitioners, test-takers) with or without programming skills to compute the exact normative scores. These tools include: a) a tool for generating a publication-ready, human-readable report in a typical setting (R GAMLSS model with several norm-predictors, of which several are potentially modelled using P-splines); b) a tool for generating machine-readable object, which may be used by norms authors for publishing norms (e.g., as supplementary to the norms paper) or by anyone to develop an app that will (by using accompanying methods) support exact norm calculations to the end-users with no programming experience; c) a tool to enable the authors of the norms with little programming experience to easily generate a web app, which enables exact calculation of normative values to the end-user with no programming skills. The reporting for purposes other than to allow the exact normative evaluation is not addressed herein. Thereby, we do not explain the process or details about fitting the underlying regression model, which requires reporting additional details about the data-preprocessing, the underlying data quality, model fitting, model selection and model diagnostics.

### Construction of regression-based norms using GAMLSS

In this section we briefly outline the GAMLSS and explain how it can be used to construct the norms. While a detailed description of how to fit these models and use them in regression-based norming is beyond the scope of this paper, here we only summarize what is needed to understand our proposed recommendations and tools. A GAMLSS has been described in detail elsewhere [23–25]. A tutorial on how to use GAMLSS for regression-based norming is provided by Timmerman et al. [10].

GAMLSS enables the modeling of four parameters of a distribution (not necessarily a member of the exponential family [26]): *mu*, *sigma*, *nu*, and *tau*, related to location, variation, skewness, and kurtosis, respectively, as functions of the norm-predictors. While some distributions are completely characterized by only some of the four parameters (e.g., the Gaussian distribution that only requires *mu* (the mean) and *sigma* (the standard deviation)), the distributions commonly used in regression-based norming (e.g., the Box-Cox Power Exponential distribution) depend on all four parameters [24]. An appropriate link function relating the distribution parameters to the norm-predictors needs to be chosen to ensure that the predictions for the modeled parameter are within the range of values that the parameter can take (e.g., for the Gaussian distribution the *sigma* parameter needs to be positive, hence the link function needs to be some monotonic function that takes a positive value as an argument and returns a real number). The *gamlss* R library offers reasonable default specifications of the link functions for each available distribution, for example, for the Gaussian distribution the default link functions are the identity and the natural logarithm functions for *mu* and *sigma*, respectively.

Regression splines are a commonly used approach to model non-linear associations (see Perpreroglou et al. [17] for an overview of using splines to model non-linear associations with a focus on the R software). P-splines attempt to overcome some issues of the other types of splines (e.g., the dependence on the number and position of the knots – the points within the data range where adjacent smooth functional pieces (usually low-order polynomials used to fit the data between two consecutive knots) join each other). P-splines generally use cubic B-spline basis with many equidistant knots and a penalty term that reduces the potential problem of overfitting due to many knots [27,28]. The amount of penalty is controlled by the smoothing parameter. When constructing the norms, the Generalized Akaike Information Criterion is commonly used to determine the optimal amount of smoothing [29].

The fitted GAMLSS model can be used to construct the norms by estimating the parameters of the assumed distribution for the particular values of the norm-predictors and evaluating the distribution function (i.e., the probability of exceeding a particular value) or its inverse, the quantile function (more details are given in the Supplementary material).

### Recommendations for reporting regression-based norms

Since the first step in regression-based norming is to fit the underlying regression model, the research paper should present sufficient information needed to fully reproduce the analysis (e.g., describe in detail how the model selection was performed) and details about the model diagnostics and the underlying data quality (which includes all the data pre-processing and data cleaning steps that were performed prior to model fitting). Describing this in more detail is, however, beyond the scope of the paper, see Rigby and Stasinopoulos [30] for a detailed description. Additionally, a paper that uses GAMLSS for regression-based norming is recommended to contain the following information, in the form of supplementary material.

1. Further details about the fitted model, in the form of a table and as a computer-readable object (e.g., as an R object), that contain the following information.

1. The family used to model the outcome (e.g., the Box-Cox Power Exponential distribution).
2. Link functions used to model each parameter of the distribution (e.g., the identity function, the natural logarithm function, the identity function and the natural logarithm function, for the *mu*, *sigma*, *nu,* and *tau* parameters of the Box-Cox Power Exponential distribution, respectively).
3. The estimated linear coefficients for each parameter.
4. If the model includes additive terms, the corresponding estimated coefficients and further details required to estimate the parameters of the assumed distribution (e.g., the estimated penalized coefficients and further details needed to completely recreate the B-spline basis of the P-spline for the parameters where the additive terms are present).

2. A fully functioning web application enabling those without programming knowledge to use the published norms.

The tabulated information will enable the readers to better comprehend the underlying model and will make it easier to understand the differences between the published models. Researchers familiar with computer programming, e.g., R, will be able to use the computer-readable object for the exact evaluation of the test-taker’s score. However, since this requires computer programming skills, a fully functioning web application enabling those without any programming knowledge to use the published norms should be designed and published. We developed the necessary tools to represent the fitted GAMLSS model according to the above recommendations. These tools are described in detail in the next section.

### Tools for publishing the norms

The tools that can be used to publish the norms in accordance with the recommendations from the previous Section are available as the R package *gamlssReport* published on GitHub (rokblagus/gamlssReport). The R package is easy to install in R via *install_github(“rokblagus/gamlssReport”)*.

There are two main functions:

1. the function *gamlssReport* extracts all the necessary information from the fitted GAMLSS and represents it in the Table format and as an R object;
2. the function *ShinyApp.gamlssReport* builds the web application.

Our implementation currently allows multiple additive terms modeled by using P-splines with equidistant knots but can otherwise handle fitted models with varying complexity. P-splines are implemented since their simplicity and flexibility allows, in a powerful combination with GAMLSS, their utilization in most practical applications [18]. The package also contains other functions (e.g., *centile.gamlssReport*), which can be used to evaluate the test-taker’s score by only requiring the output of the function *gamlssReport*. The package functionality, including an illustration of how to use it in practice, is presented in the next section.

### An example

We illustrate how to use the package by providing an example. We provide all the necessary R code, appearing after the R> symbol, that is required to present the fitted model in accordance with the recommendations or to evaluate the test-taker’s score within R. We assume that the users of our package are familiar with fitting GAMLSS using the R package *gamlss* (a tutorial about using the R package *gamlss* is given in Bann et al. [31]). The analysis for our illustration was performed in R (using R version 3.6.3 [32]).

The illustration is based on the GAMLSS-based normative regression model for the standing long jump (SLJ) performance of boys published by Ortega et al. [7]. Ortega et al. utilized GAMLSS to develop reference values for health-related fitness in European children and adolescents aged 6–18 years using the FitBack dataset. The FitBack dataset includes 1,383,773 SLJ test results on children and adolscents from 31 European countries [7]. The details about pre-processing steps, model fitting, model selection, and model diagnostics are given in the original publication (see Ortega et al. [7]), here we only report the information necessary to follow the worked example. Briefly, GAMLSS was fitted assuming Box-Cox *t* distribution, modeling all four parameters of the distribution as a non-linear function of age, using P-splines, optimizing the smoothing parameter using the Schwarz Bayesian criterion [33]. Power transformation was used for *age* before including it in the model (i.e., *nage* = *age^1/2^* was included in the model).

After fitting the model, stored in R as an R object *fit*, all the necessary information required to evaluate test-taker’s score, is obtained by using the function *gamlssReport* using the object created by the GAMLSS package as the argument:

R > obj < - *gamlssReport(fit)*

The function *print* using the object generated by the function *gamlssReport* then displays the model in the table format:

*R> print(obj)*

**Fig 1:**
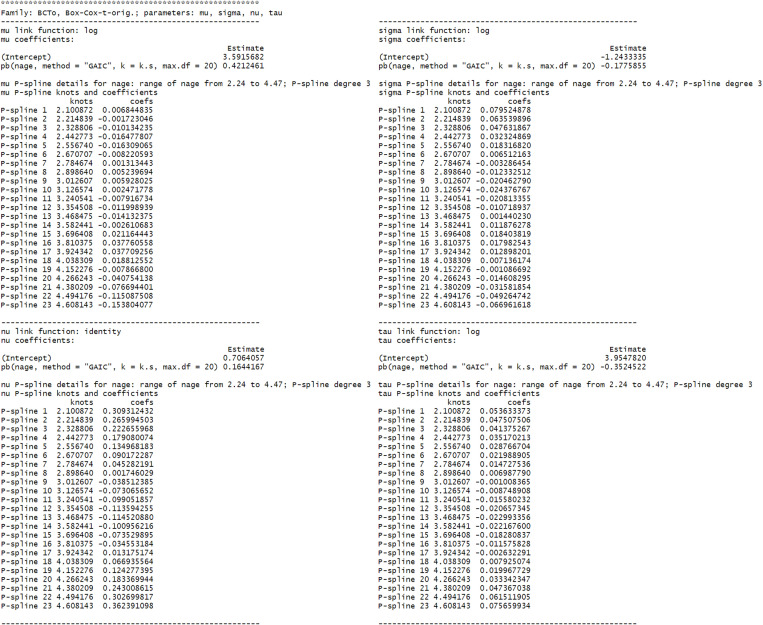
Fitted GAMLSS model for the FitBack data; Standing Long Jump test (cm) for boys.

For our FitBack example, the printed object is represented in Fig 1.

Fig 1 first reports the assumed distribution and the list of its parameters. Then there are four blocks of results, one for each parameter. In each block, the link function used for modeling a certain parameter (e.g., log for the *mu* parameter), the linear coefficients (e.g., 3.60 for the *intercept* and 0.42 for the transformed age – *nage* for the *mu* parameter), the range of the variable used in P-splines (e.g., 2.24 to 4.47 for *nage* for the *mu* parameter) and the degree of the polynomial used when forming the spline (e.g., 3 for the *mu* parameter), the knots (e.g., 2.10,...,4.61 for the 23 knots for the *mu* parameter) and their respective penalized coefficients (e.g., 0.01,...,-0.15 for the 20 + 3 = 23 penalized coefficients for the *mu* parameter) are reported. While the penalized coefficients cannot be directly interpreted, they are vital for estimating the parameters of the fitted distribution, which is required to evaluate the test-taker‘s score as illustrated in detail in the Supplementary material.

We can use the function *centile.gamlssReport* to calculate the centile for a 10-year-old boy whose SLJ was 140 cm:

*R> centile.gamlssReport(obj,y=140,newdata=data.frame(“nage”=sqrt(10)))*

The function *centile.gamlssReport* takes the object obtained by the function *gamlssReport* as the first argument, score(s) for which the centile(s) is (are) to be calculated as the second argument, and a data frame containing the values of all the norm-predictors for which the centiles are to be calculated (in our example we only need to supply the value *10*^*(1/2)*^ for *nage*, the sole norm-predictor in our model) as the final argument. In our example, the function *centile.gamlssReport* returns the value 54.7 (i.e., the boy‘s score corresponds to 54.7th centile). It is also possible to obtain the estimated parameters of the assumed distribution required to calculate the centile, using the function *predict* (see Supplementary material for more details). Note that exact evaluation of this boy’s score is not possible using only the results presented in Ortega et al. [7]. Namely, we can learn from Table 3 reported in Ortega et al. [7] that for boys aged 10.0–10.9 years, 45^th^ and 50^th^ centiles correspond to scores 135.3 cm and 141.1 cm, respectively, from where we could incorrectly assume that the centile corresponding to our boy’s score is somewhere between 45 and 50 (remember that the correct centile is 54.7). The reason for this discrepancy is that the results reported in Ortega et al. are given for the midpoint of each age interval (i.e., the scores 135.3 cm and 141.1 cm referred to earlier are the 45^th^ and 50^th^ centiles for boys aged 10.5 years). If we repeat the above calculation for a boy aged exactly 10.5 years:

*R> centile.gamlssReport(obj,y=140,newdata=data.frame(“nage”=sqrt(10.5)))*

we obtain a centile value of 48.0, which is in line with the results reported in Ortega et al. Similarly, using the summary displayed in Figure 1 in Ortega et al., we can only conclude that our 10-year-old boy’s score is between the 50^th^ and 75^th^ centile, which is likely too inaccurate to be of any practical use. While this example is not meant as a comprehensive empirical verification of the proposed recommendations against existing practices, it clearly illustrates the issues when using only select tabulated centiles for the selected values of norm-predictors or scores as a function of a selected norm-predictor for select graphically presented centiles. Despite this pitfall, the information provided currently, especially the graphical presentation, is helpful to understand general associations. Hence, we do not advise against using it, however, it should be supplemented with the information as recommended in this paper to be useful for the exact evaluation of the scores. To see which score corresponds to, e.g., the 90th centile for 10-year-old boys we can use the function *score.gamlssReport*:

*R> score.gamlssReport(obj,centile=90,newdata=data.frame(“nage”=sqrt(10)))*

The function *score.gamlssReport* has similar arguments as the function *centile.gamlssReport,* but instead of the *score* it takes the argument *centile* which represents the centile(s) for which to calculate the score(s). In our example the function returns 166.4, meaning that the 90th centile for 10-year-old boys on SLJ is 166.4 cm.

**Fig 2:**
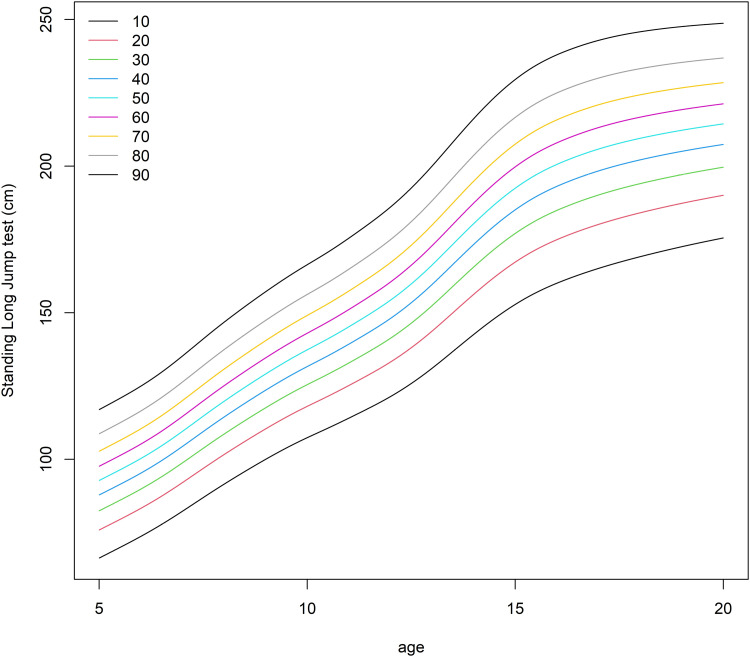
The centile curves produced by the function plot, using only the object generated by the function *gamlssReport.*

Another useful function in our package is the *plot* function, which plots the centile curves. This function extends the function *centiles* from the *gamlss* R library by allowing multiple norm-predictors when fitting the model (in the function *centiles* only one norm-predictor is supported). When there are more norm-predictors in the model, one norm-predictor for which the centile curves will be displayed needs to be chosen (this is set via the argument *xname* in the function *plot*) while the other norm-predictors are set to some value (e.g., to their respective mean or mode), which is controlled by the argument *newdata*. Using this function for our SLJ example:

R> plot(obj,xname = “nage”,range.x=obj$range.x$mu$nage,x.transform=function(x) x**2,centiles=seq(from=10,to=90,by=10),xlab=”age”,ylab = ”Standing Long Jump test (cm)”)

yields the plot presented in Fig 2. The function enables transforming the *x*-axis so that the centile curves are represented on the original scale when using the power transformation (in the above example we show the centiles as a function of *age* and not *nage*).

To produce a fully functional web app, the authors of the norms can use the function *ShinyApp.gamlssReport*. If the authors want the app to be publicly accessible, the app should be uploaded to a server that supports Shiny, for example, https://www.shinyapps.io/.

**Fig 3:**
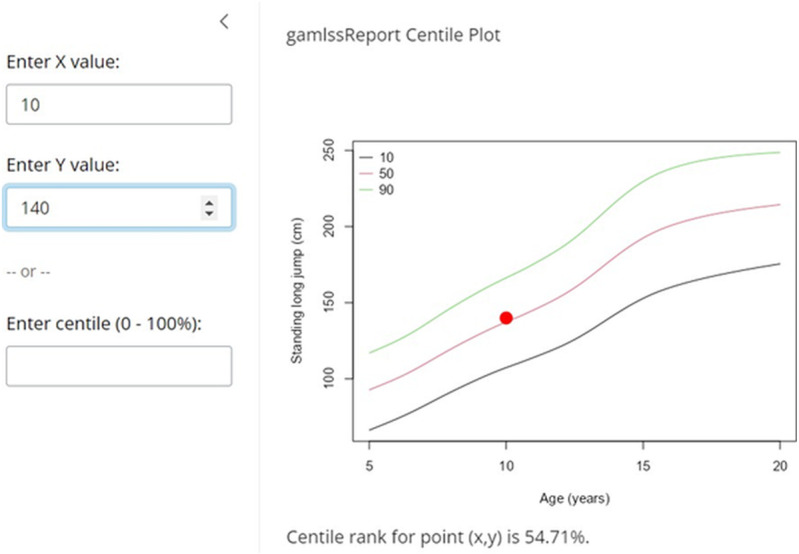
Shinny app for the FitBack data; Standing Long Jump test (cm), boys. The example shown is for evaluating the score of a 10-year-old boy with a score of 140 cm (red point).

Using the function *ShinyApp.gamlssReport* for our example produces an app which is displayed in Fig 3. This app can be used to calculate the centile by entering the *age* and the *score* (in our example we set the *age* to 10 and the *score* to 140 cm in which case the app evaluates the centile) or the score by entering the *age* and the *centile* (in which case the app would report the score at the given *centile* bellow the plot). It is not difficult to use the functions available in our R package and the R package *Shiny* to produce more complex web-based model summaries such as the one published on https://leska.shinyapps.io/FitBack/ where we summarize the norms for all the tests from the ALPHA-fit battery for both genders that were published in Ortega et al. [7].

## Discussion

We provide recommendations for publishing the regression-based norms that enable the exact interpretation (exact centile for any combination of norm-predictors) of an individual observation evaluated, without sharing any potentially sensitive information that is protected by law (e.g., GDPR in the European Union) and/or study participant’s informed consent agreement. Further, it enables a comparison of different published models by showing all the details of the fitted model (i.e., the estimated coefficients). In this manuscript, we have developed, described, and made freely available the necessary tools to publish the norms in accordance with these recommendations, using R language for statistical computing.

We illustrated the tools using the regression-based normative model published in Ortega et al. [7]. Using the published model, we showed the importance of following our recommendations. Specifically, we showed that the current practice of only reporting select centiles for selected values of the norm-predictors (or their intervals) is insufficient to exactly evaluate test-takers’ scores and, as illustrated, can lead to misleading conclusions. Furthermore, reporting (selected) centiles only for selected combinations of norm-predictors (or their intervals) contradicts the primary goal of continuous norming where one explicitly uses the information provided by the continuous, or ordered, nature of the norm-predictors in computing the norms [10]. While our example cannot be understood as a comprehensive empirical evaluation of our proposed recommendations, it clearly highlights the pitfalls of current reporting practice. Our R package currently supports additive terms (potentially many) that were modelled using P-splines (but otherwise supports the fitted models with various complexity). The P-splines in combination with GAMLSS provide one of the most powerful tools in modern regression analysis [18], and due to their flexibility reasonable results should be obtained in most practical applications. However, our proposed recommendations are general and apply also to models where other techniques would be used to model non-linear associations: in all cases the researchers are recommended to report the entire fitted model including the details about the fitted non-linear associations. For example, if the model was fitted using fractional polynomials [34], the authors are recommended to report, according to point 4 of our recommendations, for every fractional polynomial (usually one, two, or three are used for each norm-predictor), its respective power in which the norm-predictor was raised (usually this is determined from some pre-specified list), the corresponding estimated regression coefficient, and further details about potential transformation of the norm-predictors (e.g., in the R *gamlss* library the norm-predictor is shifted and scaled). The package’s functionality will be enhanced in the future allowing also other types of non-linear terms (e.g., cubic splines, fractional polynomials).

The paper only addressed the reporting recommendations for the purpose of using the norms to precisely evaluate test-takers’ scores but neither addressed nor described how to fit the underlying regression model. There is sufficient literature on how to fit these models (which involves data cleaning, model selection, and model diagnostics) and how to present sufficient information to enable complete reproducibility of the analysis, relevant references and examples are provided throughout this paper. While describing this in detail is beyond the scope of our paper, it should be self-evident that disclosing this valuable information is also necessary.

By following the recommendations presented herein, the authors of the regression-based norms will also enable others to use these results in future research that necessitates high-quality norms for norm-referenced tests. For example, Radulović et al. [35] used the normative model published by Blagus et al. [6] to investigate the secular trends in physical fitness, while Martinko et al. [36] used these regression-based norms to investigate weight-based disparities in physical fitness. None of these studies would have been possible without access to the underlying regression model. Following the recommendations presented, anyone will be able to access these models while data privacy is maintained, since the original data used to fit the models do not need to be shared.

## Conclusions

The main conclusions can be summarized as follows.

1. The paper calls for the abolition of the current practice of publishing regression-based norms, which contradicts existing principles of open science and FAIR [37].
2. The paper recommends that the underlying regression models are fully disclosed with all the details published in human and machine-readable form, complemented with the tools that will enable experts and test-takers to evaluate test scores easily and exactly. Published models should avoid publishing personal data, on which norms were constructed, so that they can be shared without limitations.
3. The paper provides tools for publishing and using norms for the case of P-splines and *gamlss* R library, probably the most flexible and frequently used methodologies for constructing norms, which in most cases enables accurate prediction of test-taker’s scores. The tools also avoid the need for publishing any personal data along with the norms (which is a limitation of the *gamlss* library).
4. The paper presents an example for the authors of current and future norms, which simplifies and accelerates the process of publishing norms by using the tools inside the *gamlssReport* library, and in line with the recommendations.

## Supporting information

S1 File The file contains additional details about using the fitted model to evaluate the test-taker’s score, details about the application to the FitBack dataset, and some additional technical details. (PDF)
